# Deficiency of transcription factor Nkx6.1 does not prevent insulin secretion in INS-1E cells

**DOI:** 10.1038/s41598-023-27985-7

**Published:** 2023-01-13

**Authors:** Vojtěch Pavluch, Hana Engstová, Jitka Špačková, Petr Ježek

**Affiliations:** Department of Mitochondrial Physiology, No. 75, Institute of Physiology of the Czech Academy of Sciences, Vídeňská 1083, Prague, 14220 Czech Republic

**Keywords:** Cell biology, Physiology, Endocrinology

## Abstract

Pancreatic-β-cell-specifying transcription factor Nkx6.1, indispensable for embryonic development of the pancreatic epithelium and commitment to β-cell lineage, directly controls the expression of a glucose transporter (*Glut2*), pyruvate carboxylase (*Pcx*), and genes for insulin processing (endoplasmic reticulum oxidoreductase-1β, *Ero1lb*; zinc transporter-8, *Slc30a8*). The Nkx6.1 decline in aging diabetic Goto-Kakizaki rats contributes to β-cell *trans*-differentiation into δ-cells. Elucidating further Nkx6.1 roles, we studied Nkx6.1 ablation in rat INS-1E cells, prepared by CRISPR/Cas9 gene editing from single colonies. INS-1E^Nkx6.1**–**/**–**^ cells exhibited unchanged glucose-stimulated insulin secretion (GSIS), moderately decreased phosphorylating/non-phosphorylating respiration ratios at high glucose; unchanged but delayed ATP-elevation responses to glucose; delayed uptake of fluorescent glucose analog, but slightly improved cytosolic Ca^2+^-oscillations, induced by glucose; despite approximately halved *Glut2*, *Pcx*, *Ero1lb*, and *Slc30a8* expression, and reduced nuclear receptors *Nr4a1* and *Nr4a3*. Thus, ATP synthesis was time-compensated, despite the delayed GLUT2-mediated glucose uptake and crippled pyruvate-malate redox shuttle (owing to the PCX-deficiency) in INS-1E^Nkx6.1**–**/**–**^ cells. Nkx6.1 thus controls the expression of genes that are not essential for acute insulin secretion, the function of which can be compensated for. Considerations that Nkx6.1 deficiency is an ultimate determinant of β-cell pathology beyond cell trans-(de-)differentiation or β-cell identity are not supported by our results.

## Introduction

Recent advances in studies of the etiology of type 2 diabetes focus on a consensus, pointing out to the “altered β-cell identity” as a concept describing processes in type 2 diabetes development in humans^1^, in contrast to concepts^2^ of pancreatic β-cell de-differentiation and/or *trans*-differentiation, which were previously considered to be essential causes^1–10^. The maintained β-cell identity allows an altered transcriptome and metabolome, but only when the main minimum functional features of pancreatic β-cells are conserved. The precise number and nature of these minimum features is currently unknown.

Pancreatic β-cell dedifferentiation denotes a regression toward an earlier progenitor state with a stem-cell-like phenotype, whereas *trans*differentiation means a change into other islets cell types, mostly to α-cells or δ-cells^1–10^. Progress in our understanding of the transcription factors controlling rodent embryonic development of the pancreatic epithelium and commitment to β-cell lineage identified several transcription factors as “β-cell-specifying”^11,12^, such as Nkx6.1, Nkx2.2, Pdx1, FoxA2, Six2 and Six3.

Nkx6.1 is a homeobox-containing transcription factor^13^, with increased expression in the trunk area of the pancreatic epithelium from mouse embryonic days 9.5 to 13 (E9.5 to E13), and it determines the later endocrine lineage^12^. Pancreatic progenitor cell (*Pdx1*^+^/*Nkx6.1*^+^), endocrine progenitor cell (*Ngn3*^+^/*Nkx6.1*^+^), and pancreatic β-cell lineage can be recognized, i.e. those expressing the insulin gene (*Ins*), (*Ins*^+^/*Gcg*^–^/*Pdx1*^+^/*Nkx6.1*^+^ after E13)^14^. Numerous relationships between transcription factors have been reported, while details of the differentiation into functional β-cells have been discovered^4,5,10–12^.

Another sorting was performed for mice, based on expression of the *Flattop* gene (*Fltp*) and *Wnt/Pcp* effector gene^15^. Thus the predominant β-cell fraction was *Nkx6.1*^+^*Fltp*^+^, which has high insulin, high oxidative phosphorylation (OXPHOS), i.e. the mitochondrial activity essential for glucose-stimulated insulin secretion (GSIS), and higher metabolism; while exhibiting a certain specific gene expression pattern. Another fraction (up to 10%) of so-called “hub” β-cells was identified in mouse pancreatic islets, which influences the remaining “follower’’ population of β-cells^16^. These “hub” β-cells have lower insulin granule content, reduced expression of *Pdx*1 and drastically low Nkx6.1 levels; but exhibit an essential orchestrating role during glucose-induced Ca^2+^ signaling^16^. These findings demonstrated a lack of exclusivity of Nkx6.1 for β-cells. The transcription factor Nkx6.1 could be recognized, together with Nkx2.2, Pdx1, FoxA2, Six2 and Six3, as β-cell-specifying factors, however with the exception of “hub” β-cells, which have almost no Nkx6.1^15^.

Previous experiments with mice have suggested an essential role of Nkx6.1 for β-cells, claiming an indisputable Nkx6.1 requirement for β-cell homeostasis and GSIS^17,18^. GSIS was also found to be suppressed in INS-1 832/13 cells^19^. The overexpression of Nkx6.1 led to increased GSIS^17^. Nkx6.1 was reported to upregulate genes for insulin processing, such as endoplasmic reticulum (ER) oxidoreductase-1β (*Ero1lb*) and zinc transporter-8 (*Slc30a8*); glucose homeostasis genes, such as those encoding glucose transporter (*Glut2*), and pyruvate carboxylase (*Pcx*)^18^; likewise the enzyme of the last gluconeogenesis step, glucose-6-phosphatase catalytic subunit-2 (*G6pc2*)^18^. Also, some genes required for β-cell proliferation are directly induced by Nkx6.1, such as Aurora kinase A (*Aurka*, which degrades the cell cycle regulator p53)^20^; G1/S-specific cyclin-D2 (*Ccnd2*)^18^; G0/G1 switch regulatory protein-7 (*c-Fos*); and nuclear receptor subfamily-4 group A member-1 (*Nr4a1*) and 3 (*Nr4a3*)^18^. Transcription factors of β-cell development are also under the control of Nkx6.1^18^, notably *Rfx6*, *MafA*, *Mnx1*, and *Tle3*.

The suppression of Nkx6.1 typically activates neurogenin 3 (*Ngn3*) in β-cells, hence stimulating their *trans*differentiation into δ-cells^15^. This explains our observed Nkx6.1 decrease in aging diabetic Goto-Kakizaki rats in association with β-cell *trans*differentiation into δ-cells^21,22^. Nkx6.1 also competes with Pax6 for the G1 element of the glucagon (*Gcg*) promoter^23^. Nkx6.1-deficient mice reportedly have a decreased pancreatic β-cell number^24^ and a defect in nutrient-induced β-cell expansion, associated with a low expression of glucagon-like peptide-1 (GLP-1) receptor (*Glp1r*)^25^.

To further investigate the role of Nkx6.1 in the function of β-cells, we prepared rat insulinoma INS-1E cells lacking Nkx6.1 (INS-1E^Nkx6.1**–**/**–**^ cells), using CRISPR/Cas9 gene editing. We initially intended to modify this pancreatic β-cell model into a presumable model of *trans*/de-differentiation (or altered β-cell identity). However, unlike in the previous reports^18,19^, we still found efficient GSIS, equal to the control cells, whereas ATP-elevations were delayed, but reached the same maximum, and ATP synthesis intensity (the latter determined from respiration parameters) was only slightly decreased. We conclude that INS-1E^Nkx6.1**–**/**–**^ cells are not dedifferentiated β-cells, but due to their phenotype, they instead resemble the “hub” β-cells.

## Results

### Unaffected GSIS in Nkx6.1-ablated INS-1E cells

**Figure 1:**
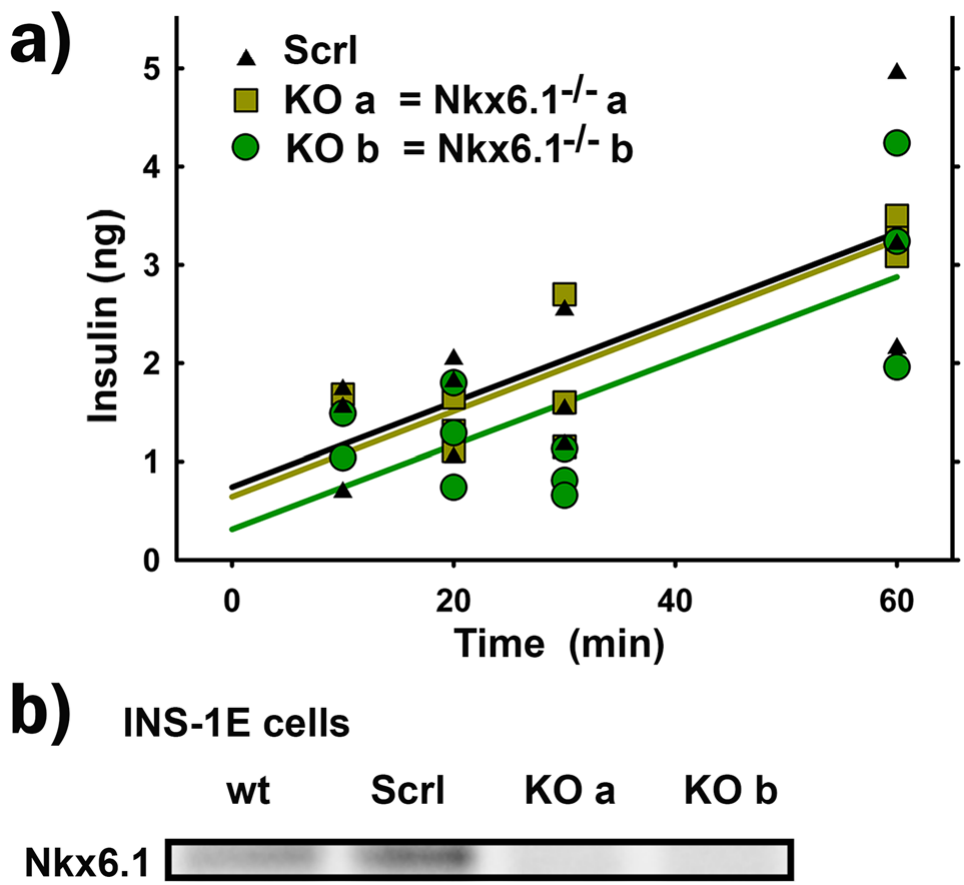
(**a**) Insulin secretion kinetics is not affected by Nkx6.1 ablation—insulin secretion was assayed in triplicates for control INS-1E^scrl/scrl^ cells (*black*) or lines (**a**) (*yellow green*) and (**b**) (*green*) of INS-1E^Nkx6.1**–**/**–**^ cells, when stimulated with 20 mM glucose adjustment after 1-h preincubation in the KRH medium, containing 3 mM glucose. Data were fit by linear regressions yielding GSIS rates of 101% ± 20% and 99% ± 30% for lines (**a**) and (**b**) relatively to INS-1E^scrl/scrl^ cells. (**b**) Nkx6.1 protein expression.

Similarly to control INS-1E^scrl/scrl^ cells (Fig. 1a), INS-1E^Nkx6.1**–**/**–**^ cells exhibited a relatively fast kinetics of insulin secretion stimulated by 20 mM glucose after 1-h *pre*-incubation in the KRH medium, containing 3 mM glucose (Fig. 1). These cells responded with a very low insulin secretion rate, when kept still at 3 mM glucose (not shown, but see Ref.^26,27,31^). When after 1-h *pre*-incubation, glucose was initially adjusted to 20 mM, a significant insulin release occurred in both control INS-1E^scrl/scrl^ cells as well as two lines, ***a*** and ***b***, of INS-1E^Nkx6.1**–**/**–**^cells (Fig. 1a). For the lack of Nkx6.1 in these two cell lines originating from single cells see Fig. 1b and Supplementary Information Fig. S1. The control INS-1E^scrl/scrl^ cells were prepared using the same CRISPR/Cas9 gene editing procedure followed by line selection from a single cell, however using a scrambled gRNA sequence. The rates of GSIS, obtained by linear regressions of data, were calculated to be 101% ± 20% and 99% ± 30% for lines ***a*** and ***b*** of INS-1E^Nkx6.1**–**/**–**^cells, respectively. The amounts of insulin released at 60 min exhibited statistically insignificant differences. Control INS-1E^scrl/scrl^ cells exhibited similar GSIS rates to parental (wt) INS-1E cells (not shown, see Refs.^26,27,31^).

### Retained glucose-induced ATP elevations in Nkx6.1-ablated INS-1E cells

**Figure 2:**
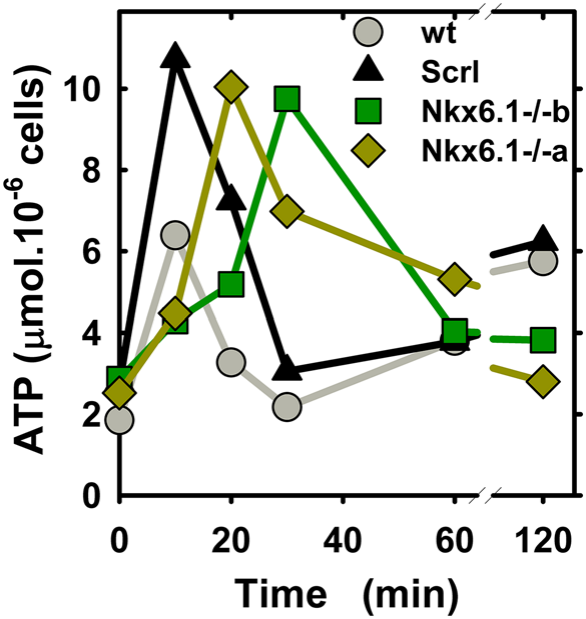
Time course of ATP elevation after 3 to 20 mM glucose adjustment—relative ATP amounts assayed by bioluminescence after adjustment of glucose from 3 to 20 mM are plotted for parental INS-1E cells (wt, *gray*), INS-1E^scrl/scrl^ cells (*black*) and line (**a**) (*yellow green*) and line (**b**) of INS-1E^Nkx6.1**–**/**–**^ cells (*green* ).

Since besides redox signaling^26^, GSIS depends strongly on OXPHOS, and hence on the elevated ATP synthesis resulting from the enhanced glucose catabolism^26–32^, we further estimated total cellular ATP levels, which increase in response to the elevated glucose. After 1-h *pre*-incubation with 3 mM glucose and subsequent adjustment of glucose to 20 mM, both INS-1E^Nkx6.1**–**/**–**^ line ***a*** and line ***b*** cells exhibited about a 10-min and 20-min delayed time course of the increase in total ATP levels, respectively, with almost unchanged amplitudes of maxima (Fig. 2; for wt parental cells, see also Ref.^31^).

### Insignificantly altered OXPHOS in Nkx6.1-ablated INS-1Ecells

**Figure 3:**
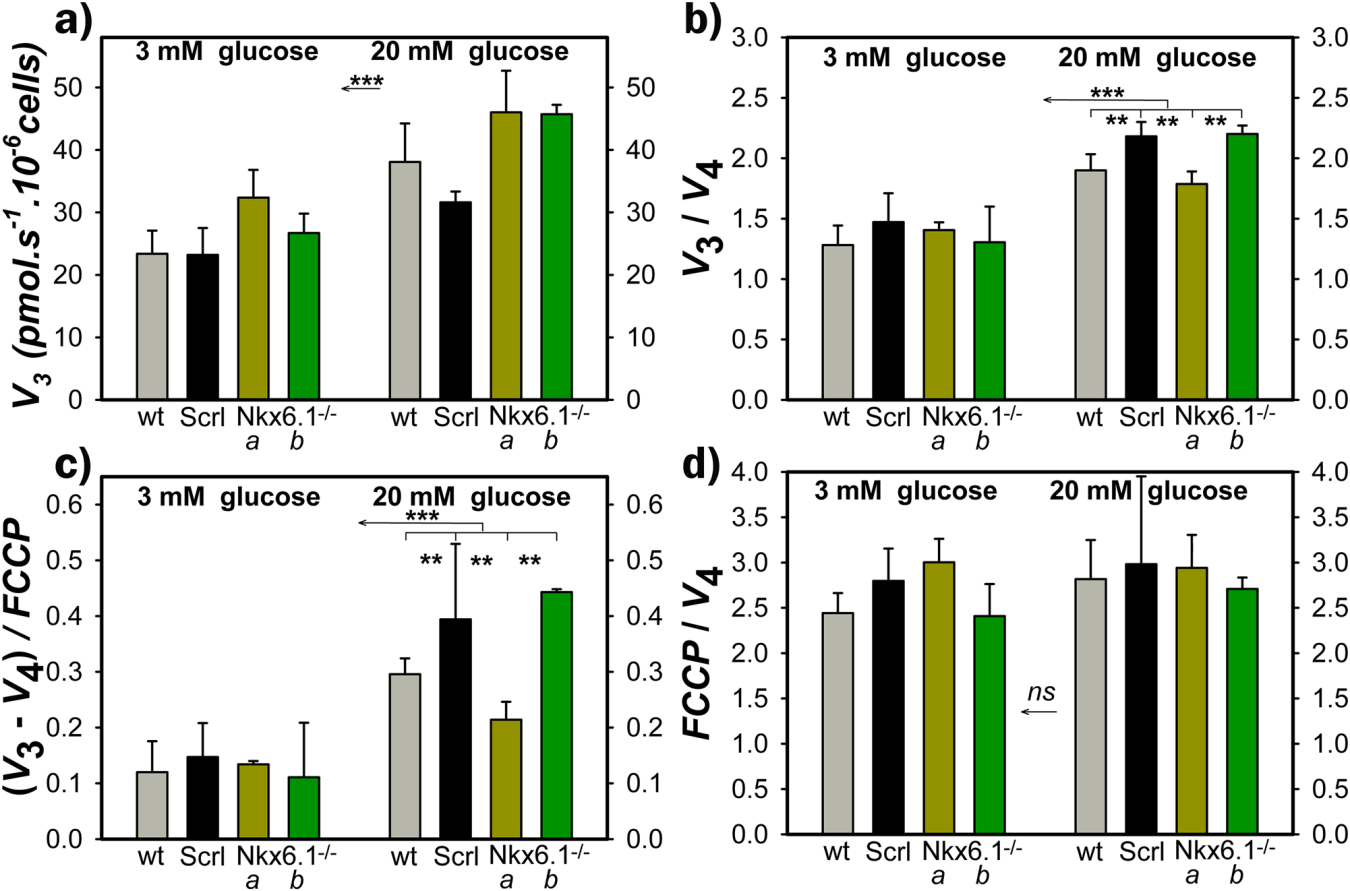
Phosphorylating respiration is not affected by Nkx6.1 ablation—panels show for wt (*gray*), INS-1E^scrl/scrl^ (*black*), line ***a*** (*green*) and ***b*** (*dark green*) of INS-1E^Nkx6.1**–**/**–**^ cells at 3 mM and 20 mM glucose: (**a**) Rates of phosphorylating (endogenous) respiration (*V*_3_); (**b**) parameters *R*r = *V*_3_/*V*_4_, i.e. ratios of *V*_3_ to non-phosphorylating respiration rates (*V*_4_), which reflect OXPHOS intensity; (**c**) parameters *A*r = (*V*_3_ **–** *V*_4_) / *FCCP*, where the *FCCP* is the uncoupled rate in the presence of uncoupler FCCP (titrated to optimum concentration yielding a maximum rate); (**d**) Ratios of *FCCP*/*V*_4_. Number of cell passages *N* was 3–6 on which *n* of 5–13 respiration assays were performed. ANOVA: ***p < 0.001; **p < 0.05.

Since the OXPHOS intensity can be *semi*-quantified from the respiratory parameters^27,31^, we further estimated the phosphorylating respiration rate (*V*_3_) (Fig. 3a), non-phosphorylating respiration rate (*V*_4_; established with oligomycin) and maximum respiration rate (*V*_max_; determined as maximum from the sequential titrations with aliquots of a mitochondrial uncoupler FCCP) (Fig. 3a–d). When calculated for average respiration rates for transitions from 3 to 20 mM glucose (number of cell samples *N* = 3–6; number of estimations *n* = 5–13), the parameters *R*r = *V*_3_/*V*_4_ increased from 1.1 ± 0.2 to 1.6 ± 0.1 and from 1.2 ± 0.5 to 2.2 ± 0.2 for the two INS-1E^Nkx6.1**–**/**–**^ lines ***a*** and ***b***, respectively, compared to the increase from 1.6 ± 0.3 to 2.1 ± 0.4 in INS-1E^scrl/scrl^ cells. A slightly diminished *R*r for line ***a***, notably its lower rise, indicates a slightly decreased intensity of ATP synthesis (OXPHOS is zero when *R*r = 1), and its reduced increase with low-to-high glucose transition. Similar data were obtained when calculated for each individual cell batch and averaged afterwards (Fig. 3b).

Next, we calculated the parameters *A*r = (*V*_3_ **–** *V*_4_)/*FCCP*, where the *FCCP* rate is equal to *V*_max_. The parameter *A*r indicates the maximum capacity of the respiratory chain, which is used for ATP synthesis. For transitions from 3 to 20 mM glucose, calculations using average rates yielded increases in parameters *A*r from 0.14 ± 0.05 to 0.19 ± 0.09, and from 0.1 ± 0.06 to 0.44 ± 0.05 for lines ***a*** and ***b***, respectively; compared to the increase from 0.13 ± 0.09 to 0.36 ± 0.17 for INS-1E^scrl/scrl^ cells. Individual calculations of *A*r gave similar data (Fig. 3c). Note that all cell lines, parental, and INS-1E^scrl/scrl^ INS-1E^Nkx6.1**–**/**–**^ lines ***a*** and ***b*** exhibited nearly identical ratios of *V*_max_ to the non-phosphorylating respiration rate *V*_4_ (Fig. 3d), which is substantiated by the existing H^+^ leak. We conclude that INS-1E^Nkx6.1**–**/**–**^ lines ***a*** and ***b*** exhibit bioenergetics parameters that are compatible with insignificantly altered ATP synthesis and reflect its increase upon the transition between 3 and 20 mM glucose.

### Delayed 6-NBD deoxyglucose uptake in Nkx6.1-ablated INS-1E cells

**Figure 4:**
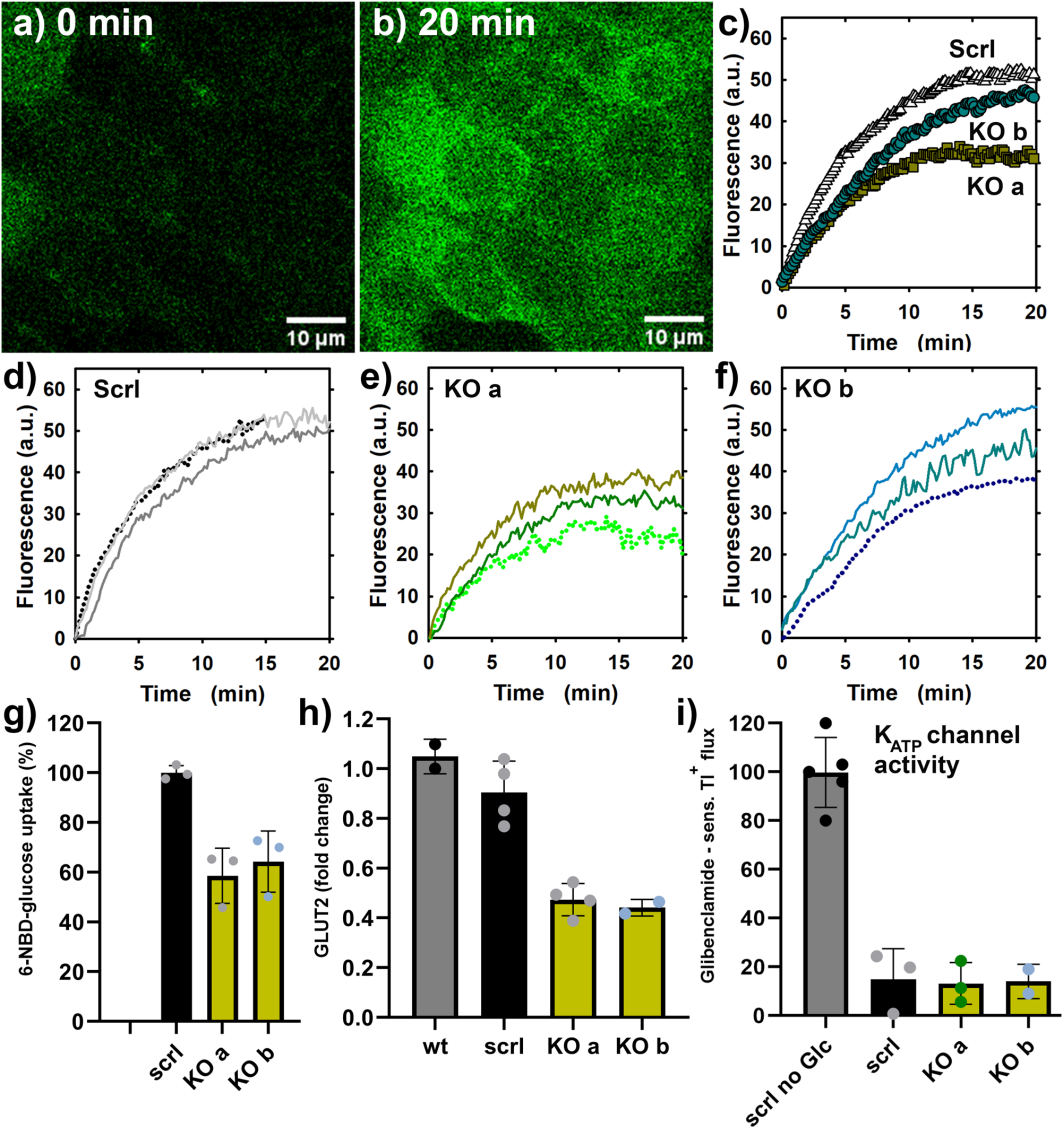
Glucose uptake is delayed Nkx6.1-ablated INS1E cells containing around half GLUT2, but intact K_ATP_ channel—(**b**–**g**) 6-(N-(7-Nitrobenz-*2-oxa-1,3-diazol-4-yl)amino)-6-d*eoxyglucose (6-NBDG) uptake into cells assessed in a confocal microscope; (**h**) relative transcripts rates of GLUT2; (**i**) Responses of Tl^+^ fluxes to glucose as surrogate for K^+^ fluxes, to which a K_ATP_ is a major contributor. Panels (**a**,**b**) shows confocal images at time zero (**a**), when wt INS-1E cells are dark and the just added 100 μM 6-NBDG causes a fluorescence background around cells; and 20 min after (**b**), when cells are filled with 6-NBDG. (**c**) Compares time-courses averaged from individual time courses, displayed on panels (**d**–**f**). Basal rates were already subtracted, i.e. those when the GLUT2 inhibitor (100 μM BAY-876) blocked the 6-NBDG uptake by up to ~ 95%. (**g**) Shows statistics of initial rates of the 6-NBDG uptake. (**h**) Shows relative *Glut*2 transcripts amounts. (**i**) Indicates no closure of K_ATP_ channels in the absence of glucose (Tl^+^ influx rate is normalized to 100%) and equal glucose-stimulated closure of K_ATP_ channels independent of the Nkx6.1 ablation.

In agreement with the previously reported Nkx6.1-mediated control of the gene for a glucose transporter (*Glut2*)^18^, we observed decreased and/or delayed transport of fluorescent glucose analog 6-(N-(7-Nitrobenz-*2-oxa-1,3-diazol-4-yl)amino)-6-d*eoxyglucose (6-NBDG, present at 100 μM) into INS-1E^Nkx6.1**–**/**–**^ cells, relative to control INS-1E^scrl/scrl^ cells (Fig. 4). *J*_6-NBDG_ rates of increases in integral fluorescence intensities in regions of interest (ROI, equal to individual cells, Fig. 4a,b) were faster in control and parental cells in contrast to slower rates in INS-1E^Nkx6.1**–**/**–**^ lines ***a*** and ***b*** (Fig. 4c–f), when the inhibited transport rates by the specific GLUT1,2 inhibitor BAY-876 were subtracted. 100 μM BAY-876 blocked *J*_6-NBDG_ rates by ~ 95%, while 25 mM glucose caused a ~ 50% inhibition of *J*_6-NBDG_ rates. Therefore, we can conclude that the recorded *J*_6-NBDG_ rates predominantly reflected the GLUT2 transport activity and were delayed by 40% in INS-1E^Nkx6.1**–**/**–**^ lines ***a*** and ***b*** (Fig. 4g) in which *Glut*2 transcript was approximately halved (Fig. 4h).

### Retained function of ATP-sensitive K^+^ channel in Nkx6.1-ablated INS-1E cells

INS-1E^Nkx6.1**–**/**–**^ cells, preincubated for 1-h in KRH with 3 mM glucose, exhibited a similar rate of the Tl^+^ influx (Tl^+^ as a surrogate for K^+^), which was completely blocked by glibenclamide (Fig. 4i). After setting glucose to 20 mM, the resulting Tl^+^ influx almost fell to that of glibenclamide in both INS-1E^Nkx6.1**–**/**–**^ and INS-1E^scrl/scrl^ cells. These results demonstrate the unaffected closure of the ATP-sensitive K^+^ channel (K_ATP_) by the Nkx6.1 deletion.

### Unchanged cytosolic Ca^2+^ oscillations during GSIS in Nkx6.1-ablated INS-1E cells

**Figure 5:**
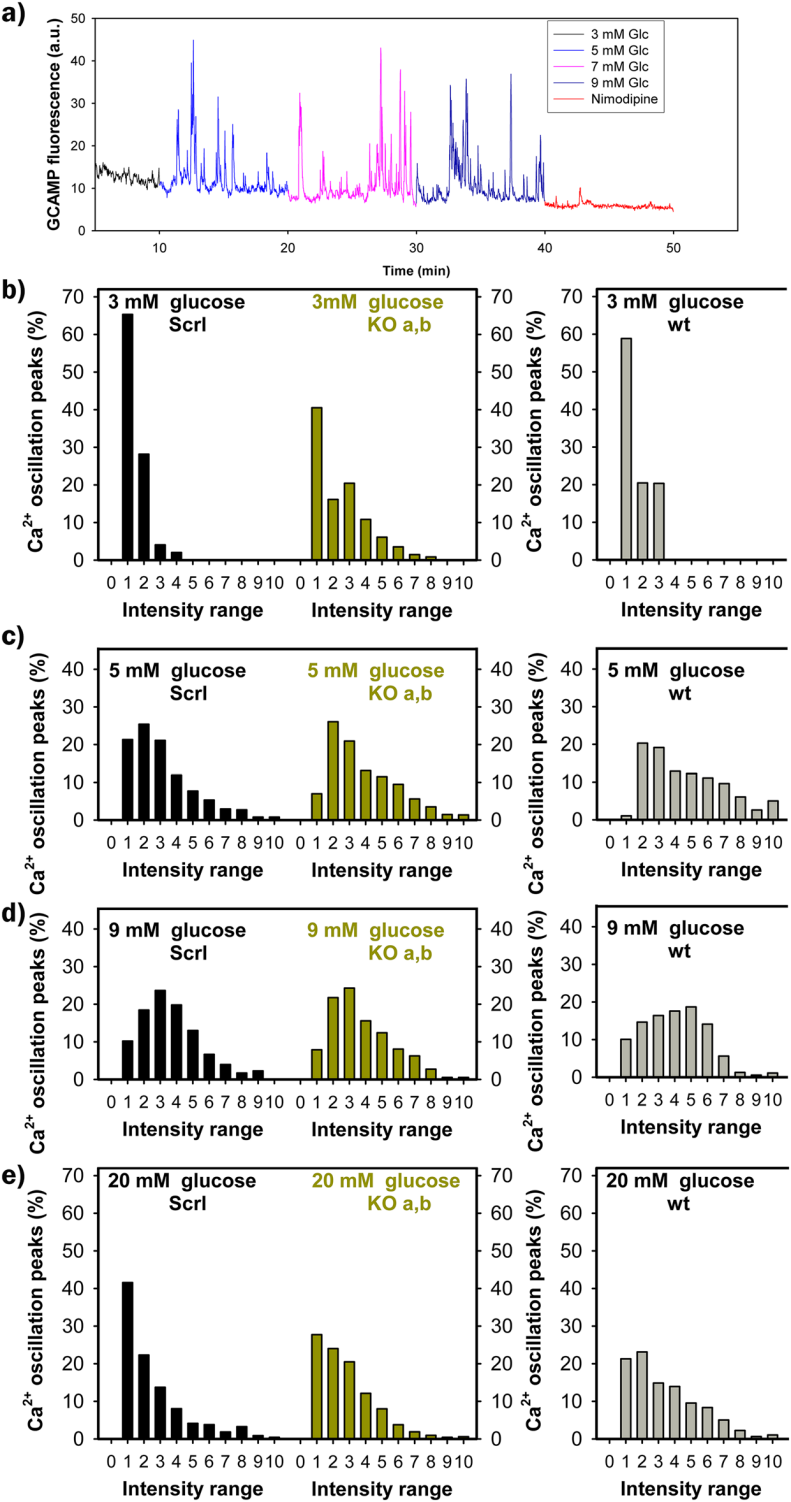
Ca^2+^ oscillations are slightly improved at 3 and 20 mM glucose in Nkx6.1-ablated INS-1E cells as surveyed by GCaMP6 fluorescence probe—(**a**) shows typical oscillations in cytosolic Ca^2+^, monitored by confocal microscopy of GCaMP6 fluorescence probe in each single cell: color-coding indicates Ca^2+^-oscillations after glucose adjustment to distinct concentrations as indicated. In the end, 5 μM nimodipine was added. (**b**–**e**) Show the corresponding histograms of intensities of Ca^2+^-oscillations, binned in deciles for INS-1E^scrl/scrl^ cells (*left, black*), grouped data of lines (**a**) and (**b**) of INS-1E^Nkx6.1**–**/**–**^ cells (*middle, green*) and wt cells (*right, gray*). Around 1000 Ca^2+^-oscillation peaks were taken into the statistics for each condition.

Since so-called “hub” β-cells, lacking Nkx6.1, were reported to likely possess an enhanced Ca^2+^-signaling ^16^, we evaluated oscillations in cytosolic Ca^2+^ at increasing glucose between 3 and 20 mM, monitored in cells with an expressed slow variant of the GCaMP6 fluorescence probe (Supplementary Information Fig. S2) by confocal microscopy (Fig. 5a). Fluorescence was collected from ROI within each single cell. However, the intensity and overall character of cytosolic Ca^2+^-oscillations was dependent on the ROI of choice, since some cells did not exhibit Ca^2+^-oscillations. Even when we did not account for an unknown noise content in the signal, cytosolic Ca^2+^-oscillations [Ca^2+^]_c_(t) exhibited very similar dependences of peak intensity histograms on glucose concentration in the responsive cells of INS-1E^Nkx6.1**–**/**–**^ and INS-1E^scrl/scrl^ cell lines as well as parental wt cells (Fig. 5b–e). However, there were distinctions in [Ca^2+^]_c_(t) histograms at 3 mM glucose, at which much higher peak intensities were encountered in both INS-1E^Nkx6.1**–**/**–**^ cell lines ***a*** and ***b***, so the histogram was significantly shifted to the right towards higher peak intensity values (Fig. 5b). Such a phenotype of excitability is natural for actual hub β-cells.

**Figure 6:**
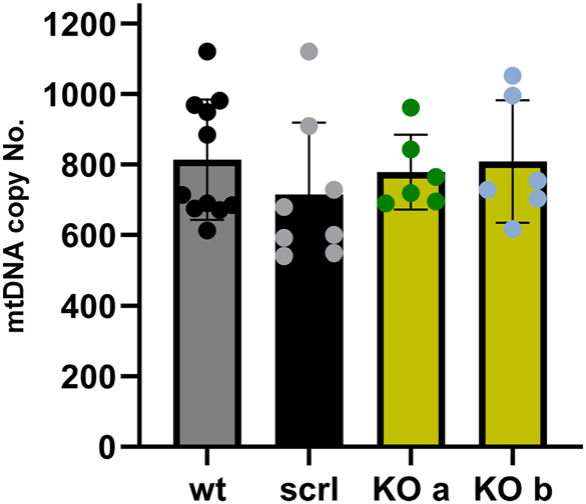
mtDNA copy number is not affected by Nkx6.1 ablation—the copy number evaluated as described in “Methods” is compared for parental INS-1E cells (wt, *gray*; number of passages *N* was 6), INS-1E^scrl/scrl^ cells (*black N* was 4) and line ***a*** (*green, green points*) and line ***b*** (*green, blue points*) of INS-1E^Nkx6.1**–**/**–**^ cells (*N* was 3 for each).

With increasing glucose concentration to 5 mM and 9 mM, [Ca^2+^]_c_(t) spikes of up to ~ 33% of the maximum intensity were the most frequent and even higher intensities were abundant (Fig. 5c,d). However, histograms for wt cells, INS-1E^scrl/scrl^ cells as well as INS-1E^Nkx6.1**–**/**–**^ cells had the same characteristics. A slight improvement in [Ca^2+^]_c_(t) oscillations was again encountered for INS-1E^Nkx6.1**–**/**–**^ cells at 20 mM glucose, at which INS-1E^scrl/scrl^ cells had almost 40% of the lowest decile intensity, whereas INS-1E^Nkx6.1**–**/**–**^ cells only had 30% and peak of higher intensity peaks were more frequently present (Fig. 6e). In all cells, 5 μM nimodipine blocked [Ca^2+^]_c_ oscillations (Fig. 5a, *red part of the trace*).

### Unaltered expression of OXPHOS-related proteins in Nkx6.1-ablated INS-1Ecells

**Figure 7:**
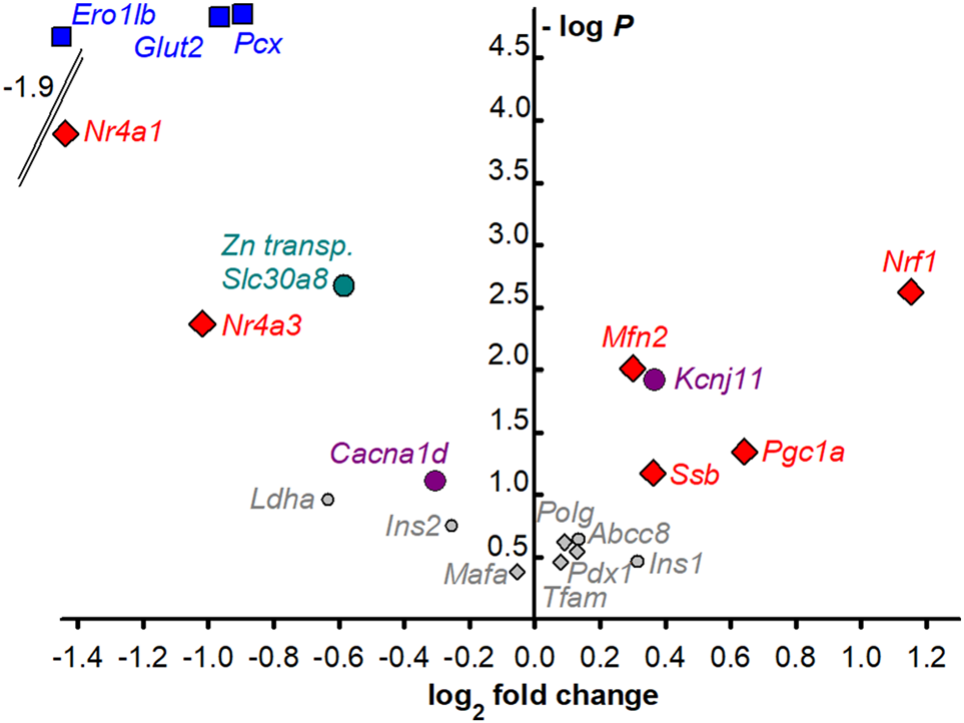
Change in selected transcripts upon Nkx6.1 ablation—a volcano plot shows increased (*right*) and decreased (*left*) transcripts of selected genes as indicated on a log2 scale, while the z-axis shows the probability of such changes (statistical significance of the assay evaluated by Student’s T-tests). Grouped data for lines ***a*** and ***b*** of INS-1E^Nkx6.1**–**/**–**^ cell are compared to INS-1E^scrl/scrl^ cells.

The moderately decreased OXPHOS in INS-1E^Nkx6.1**–**/**–**^ cells was also in accordance with findings of the unchanged copy number of mitochondrial DNA (mtDNA) (Fig. 6) and higher transcript levels of PGC1α protein (Fig. 7). The latter suggests the possibility of enhanced expression of relevant proteins under conditions, in which the expression of mtDNA-encoded subunits of respiratory chain complexes and ATP-synthase is still not saturated, hence there is no need for higher mtDNA replication.

### Pattern of transcripts for selected transcription factors and GSIS-relevant genes in Nkx6.1-ablated INS-1Ecells

Figure 7 shows a Volcano plot of changes in the transcripts of selected transcription factors and GSIS-relevant genes. Data for lines ***a*** and ***b*** were not significantly different (see examples in Fig. S3), hence the respective data were grouped together. Besides the undetectable Nkx6.1 mRNA, there is also a significantly enhanced expression of *Nrf1*, *Mfn2*, and *Pgc1a* (and slightly for *Ssb*), i.e. important factors of mitochondrial biogenesis. The transcript of the *Kcnj11* gene, which encodes the K_ATP_ channel subunit KIR6.2, was also elevated; unlike *Abcc8* encoding SUR1; whereas the transcription of the Ca_L_-encoding gene *Cacna1d* was reduced in in INS-1E^Nkx6.1**–**/**–**^ cells. The most significant decrease in transcription was found for *Pcx*, which encodes pyruvate carboxylase (Figs. S3a, 7), as well as for *Glut2*, which encodes the glucose transporter GLUT2 (Figs. 4h, 7), the *Slc30a8* gene of the zinc transporter-8, and genes encoding the nuclear receptor subfamily 4 group A member 1 and 3, i.e. *Nr4a1* (Fig. S3c) and *Nr4a3*. The most reduced expression was found for endoplasmic reticulum oxidoreductase-1β (*Ero1lb*), which was downregulated to a high probability of 26% (Figs. S3b, 7). As for the lactate dehydrogenase *Ldha*, a so-called disallowed gene in pancreatic β-cells, it remained at very low expression that was unaffected by the Nkx6.1 knockdown (Fig. 7). These data confirmed the pattern of previously established genes^18^ regulated by Nkx6.1.

## Discussion

We reinvestigated on the status of Nkx6.1 as an important transcription factor for embryonic β-cell development^12–14^ and for nutrient-induced β-cell expansion^25^. Our main conclusion is that the protein machinery required for the acute triggering mechanism of GSIS is not dependent on Nkx6.1. We demonstrated that the metabolic plasticity of INS-1E^Nkx6.1**–**/**–**^ cells is relatively wide, so that GSIS is largely maintained, due to the maintained OXPHOS. Only transients from a lower to higher intensity of ATP synthesis induced by higher glucose intake are delayed due to the Nkx6.1 deficiency and the resulting deficiency of GLUT2. This result contrasts with previous measurements^18,19^, which, however, did not assess the kinetics of the insulin release. Similarly as with the deficiency of DAPIT (a membrane F_O_-ATP-synthase subunit)^31^, this should also be partly explained by the fact that the redox signaling^26^ by NOX4-derived H_2_O_2_ cannot be suppressed by the Nkx6.1 deficiency, as reflected by the preserved normal closure of K_ATP_ channels upon the addition of glucose. Despite elevations in ATP after the initial glucose intake being delayed by ~ 10 min in INS-1E^Nkx6.1**–**/**–**^ cells *vs*. control INS-1E^scrl/scrl^ cells, the time compensated OXPHOS was sufficient for sustained GSIS.

The Nkx6.1 ablation was confirmed to reduce the GLUT2 expression^18^, resulting in a delayed glucose uptake. In accordance with the reduced *Glut2*, we demonstrated that the kinetics of glucose uptake exhibits a distinct slowdown. This was reflected by concomitantly delayed ATP elevations, induced by glucose, that however later reached sufficiently high levels that reflected high OXPHOS levels. Thus OXPHOS was time-compensated. On the other hand, a somewhat premature Ca^2+^-influx triggering at 3 mM glucose (a property of hub β-cells) and unaffected K_ATP_ channel closure-responses keep orchestrated insulin granule vesicles exocytosis and hence lead to the preserved insulin secretion kinetics. Since the time integral of the latter is rather directly proportional to the released number of insulin granule vehicles, the total amount of insulin released over 60 min as well as the kinetics of such release should not be influenced very much.

The *Pcx* deficiency^18^, i.e. decrease in pyruvate carboxylase, might be manifested as a partial blockage of the the pyruvate-malate shuttle, i.e. one of the three pyruvate redox shuttles^26^ (Fig. S4). However, compensation, by the other two shuttles might be sufficient, but disadvantageous perhaps during the early onset of type 2 diabetes. One may speculate for INS-1E^Nkx6.1**–**/**–**^ cells with a deficiency in the pyruvate-malate shuttle, that there is a faster NADH release to the cytosol^27^ and hypothetically a fractionally faster respiration (for a given pyruvate or glucose intake). This is relative to control cells, where the shuttle function disables the production of NADH by the malate dehydrogenase. Instead, transport and the cytosolic enzyme reaction sequence enables the export of redox equivalents of such NADH to the cytosol, which also decreases superoxide release into the matrix^27^. Thus speculatively, the *Pcx* deficiency might lead to higher superoxide release into the matrix and cells could be more vulnerable to oxidative stress.

Also, despite INS-1E^Nkx6.1**–**/**–**^ cells exhibiting reduced GLUT2 expression, possible expected resulting growth defects are likely to be compensated. This would proceed simply by allowing a longer development of the related phenomena, such as the glucose-stimulated expression of genes important for β-cells. Moreover, the observed enhanced expression of *Nrf1*, *Mfn2*, and *Pgc1a* (slightly for *Ssb*), which are the important factors of mitochondrial biogenesis, reflects the adaptability of INS-1E cells which lack an important regulatory transcription factor such as the Nkx6.1. Thus the enhanced mitochondrial biogenesis can balance any deteriorating effects of the Nkx6.1 deficiency. This is reflected by the unchanged mt DNA copy number in INS-1E^Nkx6.1**–**/**–**^ cells.

Despite reduction in the transcription of the Ca_L_-encoding gene *Cacna1d*, Ca^2+^-oscillations [Ca^2+^]_c_(t) exhibited the same peak intensity histograms at 5 and 9 mM glucose and slightly more intensive Ca^2+^-spikes at 20 mM glucose were found for INS-1E^Nkx6.1**–**/**–**^, as compared to INS-1E^scrl/scrl^ cells. However, these subtle differences do not entitle us to conclude that INS-1E^Nkx6.1**–**/**–**^ phenocopies hub β-cells.

As previously reported for mice with Nkx6.1 ablation^18^, genes important for the biogenesis of insulin, such as endoplasmic reticulum oxidoreductase-1β (*Ero1lb*) and zinc transporter-8 (*Slc30a8*) were also significantly reduced in INS-1E^Nkx6.1**–**/**–**^ cells. This could lead to somewhat lower total insulin storage. However, in cultured cells the concomitantly slower insulin biogenesis did not prevent the acute insulin release stimulated by glucose. Most likely this is because the amount of insulin released during GSIS in vivo accounts for only a few percent of the total stored insulin. When the triggering machinery secretes an equal amount of insulin, then only a higher relative percentage of the stored insulin granule vesicles is released. Likewise, chronically after a sufficient time given that insulin granules are still sufficiently produced in cultured INS-1E^Nkx6.1**–**/**–**^ cells. Importantly, the expression of the disallowed *Ldha* gene was kept to a minimum.

Our findings contribute to the discussion on what a β-cell is. More and more attempts to define healthy and functional pancreatic β-cell are leading to a consensus understanding that there is a spectrum of β-cell subtypes^1,10^, which is relatively dynamic and results not only from the pancreatic islet embryonic development, but also from biogenesis maintenance in the adult period. Unfortunately, distinct islet morphological organization and biogenesis in rodents *vs*. humans makes the research on rodent models less relevant for translation into human medicine. Nevertheless, easier possibilities of gene editing could justify murine studies, as we present in this work.

Bearing in mind that pancreatic islets also contain a heterologous population of β-cells also under physiological or *pre*-diabetic conditions, we need some definition^33^ of a “minimum β-cell”, which requirements should be met and what aspects are not lost, for the transformed cell to not be considered as dedifferentiated or having lost its β-cell identity. In the past, a cell was considered to be a β-cell when its transcriptome involves mRNAs for certain key β-cell transcription factors^2,17,34,35^, including the expression of *MafA*, *Neurod*1, *Foxo*1, *Pdx*1 and notably *Nkx*6.1; in addition to properly tuned *Ins* expression and “correct” machinery for GSIS and insulin secretion induced by other major stimuli, e.g. by branched-chain keto acids and fatty acids. Notably, fetal β-cells also express^36^ also *Nkx*2.2 and *Isl*1. Our data of unchanged *MafA* and *Pdx*1 not only demonstrated the independence of these factors from *Nkx*6.1, but confirmed that these should belong to the list, probably being indispensable for β-cells. In contrast, in addition to the hub β-cells, we demonstrate that *Nkx*6.1 is dispensable for acute GSIS and should not be used as a β-cell marker.

The advent of single cell transcriptomics and mass cytometry also allowed a new definition of four sub-types of human β-cells, with the predominant β1 population and less glucose-responsive β2, β3 and β4 cells^37^. These subtypes exhibit different expression^37^ of ganglioside synthase ST8SIA1 and cell surface glycoprotein CD9. The human transcriptome contains exclusively or highly expressed *PAX4*, *PDX1*, *MAFA*, *MAFB*, *DLK1*, *SIX2/3*, *ID1*, *IAPP*, *UCN3*, and *OLIG19*^38,39^, plus crucial *SIX2* and *SIX3*^40–42^.

The change in β-cell identity caused by dedifferentiation involves declines in specific proteins, including insulin, and decreasing expression of transcription factors, which were originally enriched in β-cells, together with restoring the expression of so-called β-cell disallowed genes, such as lactate dehydrogenase (LDH) and monocarboxylate transporter MCT1^11–16^. Upon dedifferentiation, expression is also elevated for transcription factors of progenitor cells that act during the original stem cell differentiation into β-cells. Thus, the expression of *Neurog*3, *Nanog*, *Pax*4 and *Sox*9 is enhanced^2^. Note that *Neurog*3 expression induces precursors of five endocrine (islet) cell types. Transcription factors specific for other islet cell types are repressed upon dedifferentiation, but restored upon *trans*-differentiation into the α-cells (*Arx*)^17^ or δ-cells (homeodomain transcription factor *Hhex* hematopoietically expressed homeobox)^43^, respectively. All these changes lead to the concomitant impairment of GSIS as a unique β-cell function, which is also caused by the reduced insulin gene (*Ins*) expression^1–10^.

Islets from type-2 diabetic patients contained a threefold elevated fraction of β-cells incapable of secreting any of the major pancreatic hormones and deficient in insulin, but expressing synaptophysin and aldehyde dehydrogenase 1 isoform A3 (ALDH1A3)^44^. Also, an increased fraction of chromogranin-A-positive cells was found in diabetic islets^45,46^. Nevertheless, other studies excluded the existence of a major β-cell de-differentiation in humans. Instead an “altered identity” was suggested as a term that better described the processes in type 2 diabetes development in humans^1^. For example, insulin-glucagon bihormonal cells exist as a 3 to 4% fraction in islets of type 2 diabetic subjects, elevated up to 16% by incretin treatment^47^. Also, an abnormal Nkx6.1 expression was found in type 2 diabetic subjects^48^. Moreover, there is a phenomenon of β-cell degranulation exists, referring to the depletion of insulin granules initiated by a metabolic stress with an unchanged transcriptome (at least transiently upon a retarded response)^44^.

## Conclusions

We confirmed that the transcription factor Nkx6.1 controls several metabolic genes, notably glucose transporter (*Glut2*) and pyruvate carboxylase (*Pcx*) genes and genes important for insulin biogenesis (*Ero1lb* and *Slc30a8*). However, when their control is dysregulated by a deficiency in Nkx6.1 that also leads to their decreased expression, OXPHOS is virtually unaffected so that the GSIS triggering is still ensured by the NOX4-mediated redox (H_2_O_2_) signaling albeit at a more slowly increasing ATP levels. Thus, the metabolic plasticity is demonstrated in cultured INS-1E^Nkx6.1**–**/**–**^ cells, as well as a moderately altered transcriptome. Consequently, certain Nkx6.1-regulated genes, previously considered to be essential for insulin secretion, can be compensated for overall integral function. Our data suggest a caution in considering Nkx6.1 deficiency to be an ultimate determinant of β-cell pathology other than stemming from cell *trans*-(de-)differentiation and changes in the β-cell identity. In conclusion, the definition of the β-cell identity is compatible with the Nkx6.1 deficiency, similarly as in the hub-β-cells.

## Methods

### CRISPR/Cas9-edited Nkx6.1 ablation in INS-1E cells

Model pancreatic β-cells, rat insulinoma INS-1E cells, were purchased from AddexBio (San Diego, CA; cat. No. C0018009). Cells were cultivated with 11 mM glucose in RPMI 1640 medium with l-glutamine, supplemented with 10 mM HEPES, 1 mM pyruvate, 5% (v/v) fetal calf serum, 50 μmol/l mercaptoethanol, 50 IU/ml penicillin, and 50 μg/ml streptomycin^21,22,26,27,31^. These parental (wt) cells were assayed after 5–15 passages*.*

CRISPR/Cas9 gene editing was performed as follows: gRNA was designed using CRISPOR, a web-based tool for genome editing experiments (http://crispor.tefor.net, version 4.6, accessed on Nov. 2018). Two different INS-1E^Nkx6.1**–**/**–**^ cell lines ***a*** and ***b*** were produced as single cell colonies, using the sgRNA sequences 5′CACCGTTGGGCGCACACAACCCGGG3′ and 5′CACCGCCAGAAGATGGGCGTCCGAC. SgRNA sequences were subcloned into the LentiCRISPR (pXPR_001) vector (kindly provided by Jakub Rohlena, Institute of Biotechnology of the Czech Academy of Sciences, Prague-West, Czech Republic) and transfected into INS-1E cells with Lipofectamine 3000 (ThermoFisher). Cells were treated with 1 μg/mL puromycin for 10 days and then cultured from single cells as separate colonies for at least three weeks to generate cell lines with deleted Nkx6.1 (*INS-1E*^*Nkx6.1****–****/****–***^* cells*, lines ***a*** and ***b*** were selected for further assays). The Nkx6.1 deletion was verified by western blot (Supplemental Information, Fig. S1). A nonsense sgRNA sequence (5′CGCACTACCAGAGCTAACTCAGATAGTACT3′) was used to repeat the above procedure in parallel as a negative control (scrambled, Scrl; *INS-1E*^*Scrl*^* cells*, were pooled from 50 single cell lines).

Prior to each experiment, cells were preincubated for 60 min (two washes of 30 min each) with 3 mM glucose, in the Krebs–Ringer HEPES (KRH) buffer (135 mM NaCl, 3.6 mM KCl, 10 mM HEPES, 0.5 mM MgCl_2_, 1.5 mM CaCl_2_, 0.5 NaH_2_PO_4_, pH 7.4).

### Insulin secretion kinetics

Cells were seeded at 0.2 10^6^ cells/well in poly-l-lysine-coated 12-well plates one day before the transient transfection and three days before the experiment. Insulin release was assayed using a Rat Insulin ELISA kit (U-E type, Shibayagi Co., Shibukawa, Japan) in a KRH buffer after 1-h of preincubation prior to glucose addition^26,27,31^.

### Elevations in total cell ATP

The quantification of ATP was performed using an ATP Assay bioluminescence kit HSII (Roche). After reaching the desired conditions, cells were mixed with boiling lysis buffer (100 mM Tris, 4 mM EDTA, pH 7.75) and further boiled for another 2 min. Samples were centrifuged at 10,000×*g* for 1 min. Diluted supernatants were mixed with luciferase reagent and a Synergy HT luminometer was used to read the bioluminescence^27,31^. To confirm that the assay procedure does not interfere with the ATP concentration determination, internal ATP standards were added to the samples during the initial experiments.

### High resolution respirometry

Routinely, an oxygraph 2k (Oroboros Instruments GmbH, Innsbruck, Austria) was used for experiments checking the respiration of INS-1E cells as described elsewhere^27,31^.

### Monitoring of uptake of fluorecent glucose analog into INS-1E cells

6-(N-(7-Nitrobenz-2-oxa-1,3-diazol-4-yl)amino)-6-deoxyglucose (6-NBDG; 100 μM) (ThermoFisher, Life Technologies Waltham, MA, USA) was used for monitoring glucose uptake into INS-1E cells. Cells were seeded onto the coverslips 48 h before the experiment and prior to confocal imaging they were incubated for 1 h in the KRH buffer with 3 mM mM glucose. 6-NBDG was added at the beginning (with no other agent or together with glucose to adjust it to 25 mM or with 100 μM BAY-876, which is both a GLUT1, but also GLUT2 inhibitor). Time-lapsed confocal monitoring was performed using a Leica TCS SP8 confocal microscope (Leica, Mannheim, Germany) with excitation at 480 and emission at 540 nm. A 2D spatial integral of the fluorescence intensity was derived from the series of confocal images within the individual cell ROIs.

### Tl^+^ uptake assay as surrogate for K^+^ uptake into INS-1E cells

Assays of Tl^+^ influx rates were performed using the FluxOR potassium ion channel assay (ThermoFisher)^26^. Since plasma membrane depolarization is caused by an inhibition of K_ATP_ channels, the cells were first preloaded with “stimulus buffer” containing Tl^+^. Upon binding cytosolic Tl^+^, the de-esterified FluxOR dye strongly increases fluorescence at its peak emission of 525 nm, which was monitored in a Shimadzu RF 5301 PC spectrofluorometer.

### GCaMP6 assay for Ca^2+^ oscillations in INS-1E cells

The vectors pGP-CMV-GCaMP6f (Plasmid #40753) and pGP-CMV-GCaMP6s (Plasmid #40755) were purchased from Addgene^49^, which express the fast-responding and slow-responding but more sensitive GCaMP6 fluorescent Ca^2+^indicator, respectively. GCaMP6 represents a synthetic fusion of green fluorescent protein (GFP), calmodulin and M13, a peptide sequence from myosin light-chain kinase^50^. Cells were transfected using Lipofectamine 2000 (ThermoFisher) for 48 h. A Leica TCS SP8 confocal microscope was employed for the time-lapsed recording of integral fluorescence intensity within the individual cells ROI with excitation at 480 and emission at 510 nm. For every second, integral fluorescence intensities *F*[Ca]_c_(*t*_i_) were collected from the image of each responding single cell in widefield and data were plotted as a time course. Typically, 10 min periods (trails) were set and in each period the glucose level was adjusted, starting from 3 mM, then typically 5, 7, 9, 11, and 20 mM glucose followed and at the end 30 mM KCl was added. Numerical 1st-derivatives were calculated to ascertain peaks of oscilations, so that a peak at time *t*_i_ occurred when the derivative of *F*[Ca]_c_(t_i_) was zero at time *t*_i_. Only those zero derivatives were selected when preceded by the positive derivative and followed by the negative derivative values, thus excluding the negative–positive derivative pairs around the zero derivative, which represent functional minima. Peaks of oscillations in each period were sorted by intensities into ranges scaled by 1/10 of maximum intensity (deciles) and histograms were plotted for each period.

### Mitochondrial DNA copy number

The mtDNA from INS-1E cells was isolated using DNA lysis buffer (10 mM Tris–Cl, 100 mM NaCl, 10 mM EDTA, 0.5% SDS pH 8.0) followed by incubation in proteinkinase K, protein salting in 6 M NaCl and the precipitation of DNA by isopropanol. This was followed by SYBR Green qPCR amplification with primers annealing on the *Slco2a1* nuclear gene (which encodes solute carrier organic anion transporter family member 2A1) and the *Nd5* mitochondrial gene (bp 11,092 to 11,191 according to Genebank sequences from The National Center for Biotechnology Information, USA)^31^. For primers see Ref.^31^. The ratio between *Nd5* amplicon and half of the nuclear amplicon amounts was taken as the mtDNA copy number *per* cell.

### RT-PCR

Total RNA was extracted from cells by acid guanidinium thiocyanate-phenol-chloroform extraction using Trizol-like buffer (0.4 M ammonium thiocyanatate, 0.8 M guanidine thiocyanate, sodium acetate 0.1 M, glycerol 5% (v/v), phenol 38% (v/v), pH 4.4). The reverse transcription was performed with a cDNA GrandScript reverse transcription kit (Tataa Biocenter, Prague, Czech Republic), using 500 ng of RNA previously quantified with a NanoDrop 2000 (ThermoFisher). The real-time polymerase chain reaction was performed with Fast Evagreen qPCR Master Mix (Biotium, Fremont, CA, USA). The PCR amplification was carried out with initial denaturation at 95 °C for 20 s, followed by 45 cycles of 95 °C for 5 s and 57 °C for 10 s and 72 °C for 20 s, followed by 5 min of terminal amplification at 72 °C, using a CFX96 cycler (Bio-Rad). The primers used were as indicated in Table 1. All of them led to a single amplicon product when the described qRT-PCR procedure was performed. Data were calculated by the 2^−∆∆CT^ method (where CT is cycle threshold and ∆∆CT is CT for the gene of interest minus CT of the internal control), having selected housekeeping genes, indicated in Table 1, as an internal control for each experimental condition.

**Table 1:** Primers employed for tested rat (r) genes—in forward (F) and reverse (R) direction.

Gene	Forward primer	Gene	Reverse primer
*r-Abcc8 197 F*	ATCTACTGGACCCTGGCCTT	*r-Abcc8 197 R*	GGCTTTACTTCCCTTGGTGTC
*r-Cacna1d 244 F*	CTTTGGTACGGACGGCTCTC	*r-Cacna1d 244 R*	GCAGGGTATTTCCCCACCAG
*r-Ero1lb 172 F*	CGCCATCAACAGCACCCTAA	*r-Ero1lb 172 R*	TGAGGAGCCCTTGTAGCCAG
*r-Glut2 245 F*	GGCATGTTTTTCTGTGCCGT	*r-Glut2 245 R*	TACTGGAAGCAGAGGGCGAT
*r-Ins1-g 100 F*	CCCAAGTCCCGTCGTGAAGT	*r-Ins1-g 100 R*	CAACCTCCAGTGCCAAGGTC
*r-Ins2-g 160 F*	GTGACCAGCTACAGTCGGAA	*r-Ins2-g 160 R*	TTCCACCAAGTGAGAACCACAA
*r-Kcnj11 206 F*	ATCAGTCCAGAGGTTGGTGC	*r-Kcnj11 206 R*	GTACCTGGGCTCTGTAGGGT
*r-Ldha 183 F*	GTGCACTAAGCGGTCCCAAA	*r-Ldha 183 R*	GGCAAGCTCATCAGCCAAGT
*r-MafA 105 F*	ATCCGACTGAAACAGAAGCGG	*r-MafA 105 R*	GCACTTCTCGCTCTCCAGAAT
*r-Mfn2 114 F*	ACCTGAATCGGCACAGAGGA	*r-Mfn2 114 R*	GCAGGGACATCTCGTTTCTAGC
*r-Nr4a1 213 F*	CCAGATGCCTCCCCTACCAA	*r-Nr4a1 213 R*	GAAGCCCGGGATCTTTTCCG
*r-Nr4a3 157 F*	GATGAACGCCCTTGTCCGAG	*r-Nr4a3 157 R*	GCTTCTGGACACGTCGATGG
*r-Nrf1 263 F*	CATCCAGACGACGCAAGCAT	*r-Nrf1 263 R*	ATGCATGAACTCCATCTGGGC
*r-Pcx 158 F*	ATCTTGCACTTGTATGAGCGGG	*r-Pcx 158 R*	GTGCCTGCATTCTCATAGCCA
*r-Pdx 152 F*	ATCCACCTCCCGGACCTTTC	*r-Pdx 152 R*	CTCCGGTTCTGCTGCGTATG
*r-Pgc1a 244 F*	GACTGGCGTCATTCAGGAGC	*r-Pgc1a 244 R*	ATGTTCGCGGGCTCATTGTT
*r-Polg 124 F*	ATGCGAATGGTCCAGCGATTT	*r-Polg 124 R*	AACAGTTCCCGAGGCTCCTT
*r-Slc30a8 99 F*	CAAGCGGCTGACATTTGGGT	*r-Slc30a8 99 R*	GCAAGGTACACCAGCACACC
*r-Ssbp1 243 F*	GTGGCGATCAGGGGACAATG	*r-Ssbp1 243 R*	CAAGAAACGCTGCGTACCAC
*r-Tfam F*	GCATGATAACAAGCCCCTGGA	*r-Tfam R*	CCAGTGTTCTTAGCACGCCC

Reference gene	Forward primer	Reference gene	Reverse primer
*r-Gusb F*, glucuronidase, beta	AACAATCGGTTGCAGGGCTT	*r-Gusb R*	TCCCATTCACCCACACAACTG

For a reference gene, rat glucuronidase beta (*r-Gusb*) was used.

### Statistical analysis

Results are presented as mean ± standard deviation (SD) for *N* biological replicates or total number of estimates (*n*). Graphs were plotted and statistical analyses were performed using SigmaPlot 6.0 and SigmaStat 3.1 (Systat-Software, San Jose, CA) and ANOVA followed by the Tukey test on the pre-validated data; or, alternatively using Prism (GraphPad Software, San Diego, CA) and an unpaired Student’s T-test (when comparing two groups) or one-way non-parametric ANOVA (Tukey test). Statistical significance was set at ***p < 0.001; **p < 0.05; *p < 0.1.

## Supplementary Information

Supplementary Figures.

## Data Availability

The data sets supporting this study are available from the corresponding author, P.J., on request.
