# Supplementary material for: Deficiency of transcription factor Nkx6.1 does not prevent insulin secretion in INS-1E cells
Source: Sci Rep. 2023 Jan 13;13:683. doi: 10.1038/s41598-023-27985-7 (PMC9839711; doi:10.1038/s41598-023-27985-7)

Supplemental information

CRISPR/Cas9 deletion of „β-cell specifying“ transcription factor Nkx6.1 in INS-1E cells does not prevent insulin secretion

Vojtěch Pavluch [1], Hana Engstová [1], Jitka Špačková [1], and Petr Ježek [1,*]

[1] Department of Mitochondrial Physiology, No. 75, Institute of Physiology of the Czech Academy of Sciences, Vídeňská 1083, Prague 14220, Czech Republic; vojtech.pavluch@fgu.cas.cz (V.P.); hana.engstova@fgu.cas.cz;

* Correspondence: jezek@biomed.cas.cz; Tel.: +420-296442760

Figure S1 a) The lack of Nkx6.1 transcript in lines *a* and *b* of Nkx6.1$^{-/-}$ cells

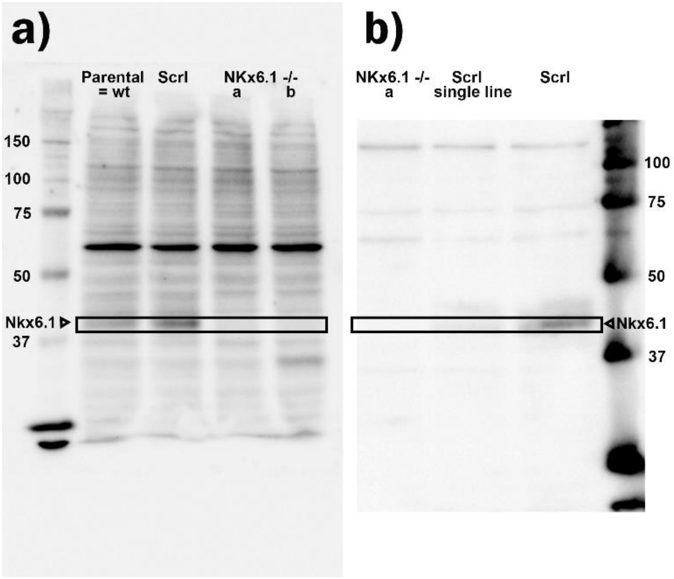

Two exemplar Western blots in panel **a**) (the square section of which was used for Figure 1b) and panel **b**) using *anti*-Nkx6.1 antibody show the Nkx6.1 protein of 44-46 kDa in wt (i.e. parental) INS1-E cells and cells pooled from 50 single-cell colonies transfected with nonsense sgRNA sequence (Scrl) as well as an example of one such a single line („Scrl single line“). Numbers describe molecular weight of the used standards.

Figure S1 b) Procedure of CRISPR/Cas9 gene editing of INS-1E cells

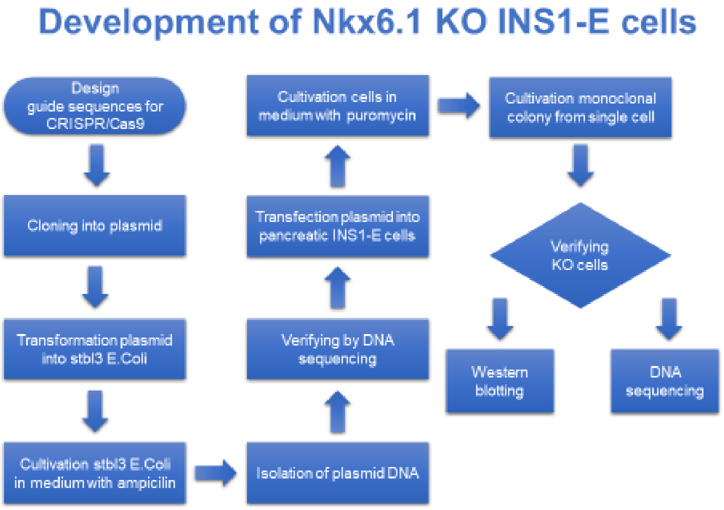

Figure S2 Exemplar expression of a slow variant of GCaMP6 fluorescence probe

Confocal microscopy image of wt INS-1E cells illustrating GCaMP6 emission at 510 nm is shown, when excited at with at 480 nm.

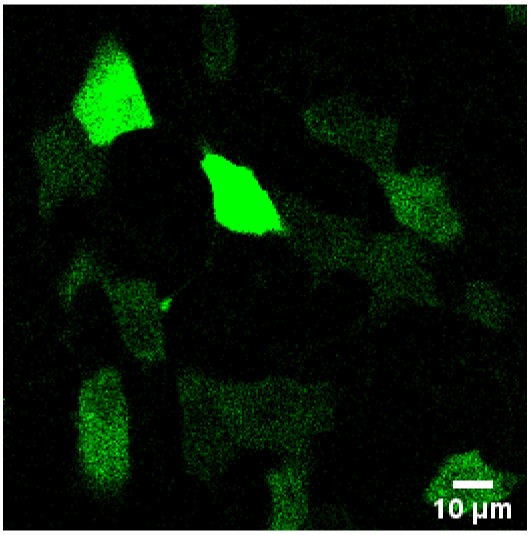

Fig.S3 Selected most pronounced changes of transcripts in Nkx6.1- ablated INS-1E cells

Panels (**a**–**c**) show the most pronounced changes in transcripts for (**a**) pyruvate carboxylase, (*Pcx*); (**b**) endoplasmic reticulum oxidoreductase-1β (*Ero1lb*); (**c**) nuclear receptor subfamily-4 group A member-1 (*Nr4a1*).

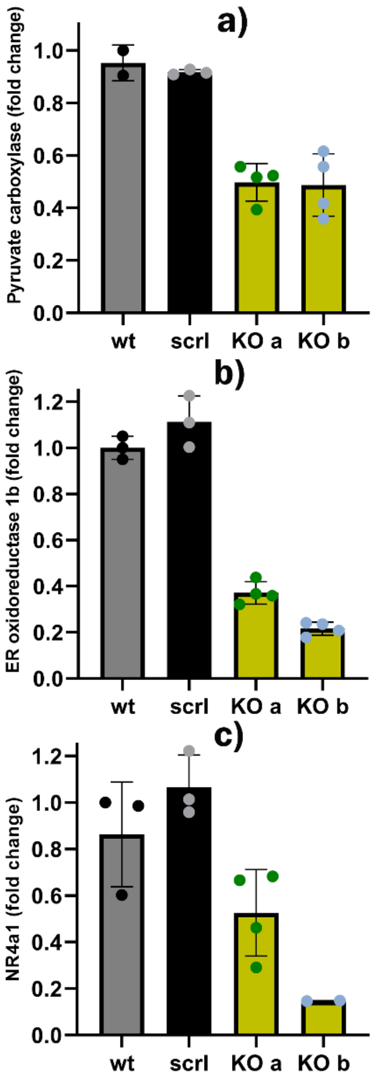

Fig.S4 Schemes of metabolic fluxes with and without participation of pyruvate carboxylase

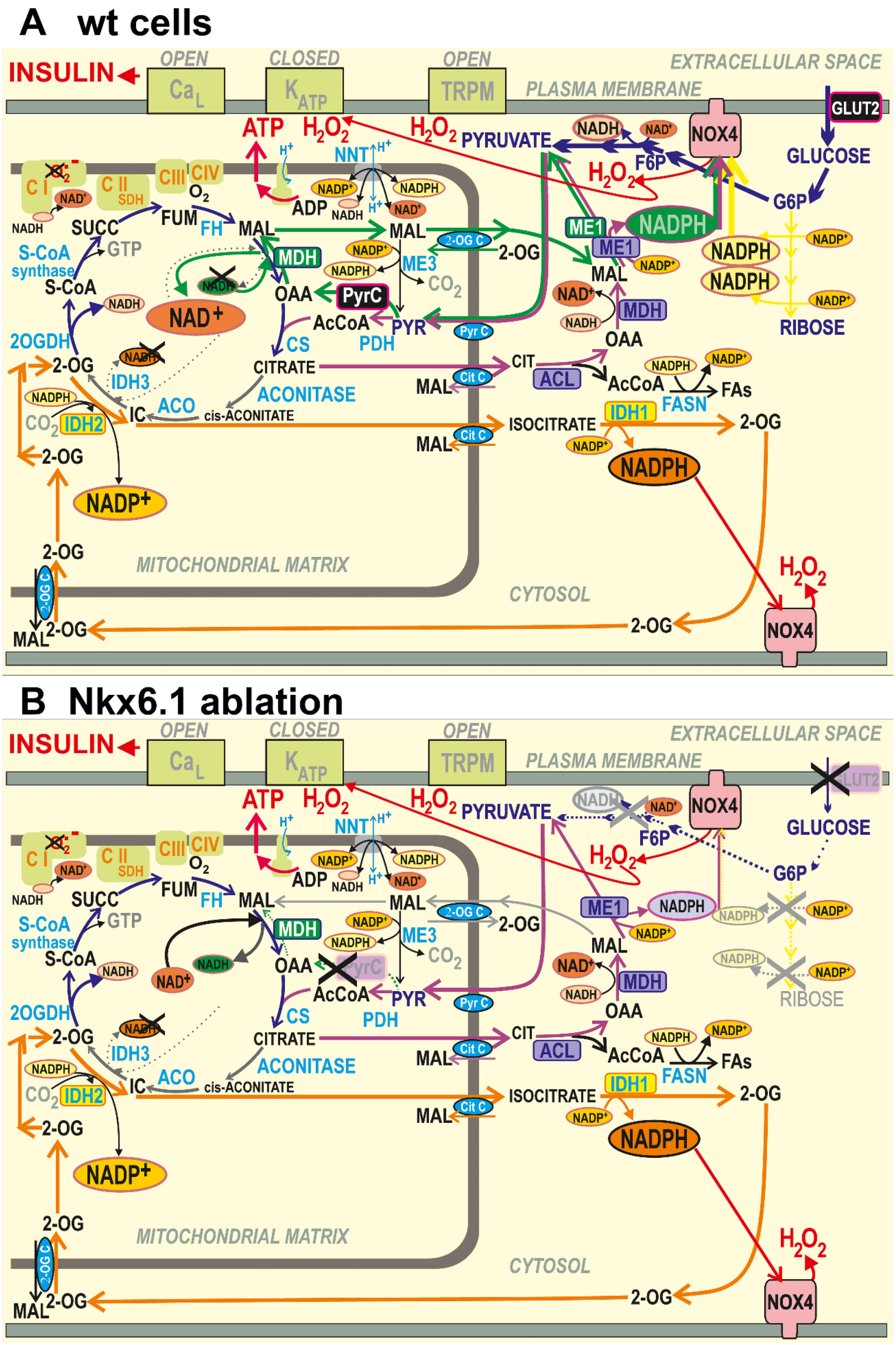

Supplement: Supplementary file 1 — Supplementary Figures. [file 41598_2023_27985_MOESM1_ESM.pdf]
